# Prevalence of Substandard Amoxicillin Oral Dosage Forms in the National Capital District of Papua New Guinea

**DOI:** 10.4269/ajtmh.20-1570

**Published:** 2021-05-17

**Authors:** Sherryl G. Robertson, Naomi T. Hehonah, Rose D. Mayaune, Beverley D. Glass

**Affiliations:** 1 Pharmacy, College of Medicine and Dentistry, James Cook University, Townsville, Australia;; 2 Division of Basic Medical Sciences, Pharmacology, University of Papua New Guinea, Port Moresby, Papua New Guinea

## Abstract

Antibiotics are commonly reported as being substandard or falsified in low- to middle-income countries, having potential to contribute to the development of antimicrobial resistance and drug-resistant infections. Amoxicillin, used to treat a number of infections and listed by the WHO as an essential medicine, presented as a good drug candidate for this study. We aimed to measure the prevalence of substandard and falsified amoxicillin oral products (tablets, capsules, and suspensions) in the National Capital District of Papua New Guinea (PNG). These oral products were surveyed in 2018 and 2019 from retail pharmacies, private and public health facilities, and the Area Medical Store, representing more than 90% of licensed medicine outlets. The product packaging was visually inspected, and the samples were analyzed for amoxicillin content using a validated high-performance liquid chromatography method. Although no falsified products were identified, 15% of the 190 products analyzed contained substandard amounts of amoxicillin. Quality varied with the dosage form (*P* = 0.002), with capsules exhibiting the lowest incidence of substandard content (4% in 2019) and tablets collected in 2018 experiencing the highest failure rate (50%). Suspension (40%) quality was compromised by failure to achieve homogeneity on reconstitution. A higher incidence of substandard content (*P* = 0.002) was associated with one major retail group. Routine testing of medicines by resource-poor countries is often unachievable, leading to the circulation of poor quality drugs, which is a global public health concern. Our study highlighted that substandard amoxicillin oral products are indeed prevalent in the NCD of PNG.

## INTRODUCTION

Combating the prevalence of poor-quality medicines has been a focus of regulatory authorities for more than two decades, with initiatives to address this issue well documented in the literature.^1–3^ Despite such efforts, poor-quality medicines remain a global health issue, with their use being associated with the development of drug resistance, patient morbidity and mortality, and a loss of confidence in health systems.^4–6^

Monitoring the quality of medicines in poorer countries is problematic because of a range of issues including insufficient controls on supply chains, inadequate pharmaceutical regulatory systems, limited financial resources, and limited laboratory capacity.^7–9^ In 2017, the WHO estimated that 10.5% of medical products in low- and middle-income countries were substandard or falsified.^10,11^ Although falsified medicines are the product of criminal intent, substandard medicines are legitimately manufactured, but fail quality specifications when tested.^10,11^ This may be the result of inadequate quality control measures during production or be caused by exposure to unfavorable environmental conditions during transport and/or storage leading to drug degradation. The most commonly reported substandard/falsified drug products in 2017 contained antimalarial (19.6%) and antibiotic (16.9%)^11^ drugs, and although the incidence may be reflective of greater screening of these essential medicines, the figures highlight the issue of poor-quality medicines to the development of antimicrobial resistance and drug-resistant infections.

Amoxicillin is listed by the WHO as an essential medicine and also classified under the Access group of antibiotics, meaning it should be available at all times.^12,13^ In oral dosage forms, amoxicillin is most commonly present as the trihydrate salt which degrades rapidly under acidic conditions in aqueous solution,^14^ whereas in the solid state, the loss of the waters of crystallization can significantly increase the susceptibility to degradation.^15^ A review^5^ of published literature (1992–2009) found that amoxicillin was the most commonly identified substandard/falsified antibiotic, detected in 29 countries. Although many reported incidents of substandard/falsified medicines are attributed to criminal activity, it has been commented^16^ that this is not always justified as drug degradation, and poor manufacturing practices are also prevalent. In tropical climates where temperature and relative humidity (RH) levels are elevated, it has been found^17–19^ that the stability of generic amoxicillin medicines can be highly variable even when maintained in their primary packaging. Stability testing (6 months, 40°C, and 75% RH) of amoxicillin potassium clavulanate tablets collected in Yemen^17^ (nine generic manufacturers) found the loss of amoxicillin content ranged from 10% to 33%. In addition, the dissolution behavior of 80% of the samples was adversely affected, leading the authors to infer that the formulations were unsuitable for storage in countries with tropical climates.^17^ Similarly, amoxicillin capsules manufactured in the Democratic Republic of the Congo showed variable degradation rates (6–24%) when subject to stability testing (6 months, 40°C, and 75% RH), leading the authors to question the validity of the 3-year shelf-life listed on the packaging.^18^ The stability of pediatric oral suspensions before reconstitution has rarely been reported; however, a study conducted in Bangladesh^19^ found eight of the 10 collected samples (80%) failed to comply with the shelf life stated on their packaging.

The dispensing of loose tablets and capsules in plastic bags, a practice common in many in low- and middle-income countries which hinders the detection of falsified medicines, is additionally problematic in tropical climates as this packaging does not afford sufficient protection against moisture.^20^ A South African study simulating local conditions in Durban (30–35°C and 75% RH) reported amoxicillin loss of 11–16% from capsules that were stored in plastic bags and flip-top bottles for 1 day.^21^

The focus of this study is Papua New Guinea (PNG), a lower-middle–income country with a population of more than seven million at the last conducted census (2011).^22^ The country experiences a high burden of communicable diseases and rapidly increasing rates of noncommunicable diseases, compounded by a high incidence of drug-resistant tuberculosis.^22^ Furthermore, 60% of the population live in areas where malaria transmission is endemic, with drug resistance first reported in 1976,^23^ and the resistance to antiretroviral drugs used as first-line treatment of HIV is rising.^24^ The Western Pacific Regional Office of the WHO has also reported that the single largest category of drug resistance in the region is to antibiotics.^24^ Papua New Guinea has no licensed pharmaceutical manufacturers^25^ and is entirely reliant upon the importation of medicines into the country. The supply chain for the private and public sectors operates independently, and previous surveys of anti-infective products have identified the presence of substandard and falsified medicines in both supply chains.^26,27^ The aim of the current study was to measure the prevalence of substandard and falsified amoxicillin products (tablets, capsules, and oral suspensions) with the view to identifying the risk factors that may contribute to the supply of these poor-quality medicines in the National Capital District (NCD) of PNG.

## MATERIALS AND METHODS

### Study design.

The location of retail pharmacies, private and public health facilities, and the Area Medical Store was identified from the PNG Ministry of Health’s listing of Registered Pharmaceutical Establishments^28^ and local knowledge. Collection of the amoxicillin products (250 mg tablets, 250 mg and 500 mg capsules, 125 mg/5 mL and 250 mg/5 mL suspensions) occurred on October 18–19, 2018 and on March 18, 2019–April 11, 2019 by volunteers trained to present as mystery shoppers.^29^ Each licensed pharmaceutical outlet was visited a number of times during the survey periods, with different volunteers purchasing alternative dosage forms. Volunteers completed an information sheet for each sample, recording the location, outlet category, and their perception of temperature at the outlet (functional air conditioning), and then stored the collected medicines in a central air-conditioned location. Product packaging was visually inspected using the “Tool for Visual Inspection of Medicines,”^30^ which documents a checklist to aid in the identification of suspicious products. All samples were subsequently air-freighted to James Cook University in Townsville Australia, stored at 5°C and analyzed for amoxicillin content within 3 months of collection. This study was approved by the School of Medicine and Health Sciences Ethics Committee at the University of PNG (UPNG) in 2018, and because of the political sensitivity surrounding medicine quality in PNG, it was agreed that the identity of the pharmacies and their affiliations would not be disclosed. Likewise, the manufacturers of the collected medicines and their brand names were to be de-identified in any publications. Sample collection from unlicensed street stalls was not permitted because of concerns regarding the safety of the volunteers.

### Chemical analysis.

Analysis was performed using methods adapted from the respective U.S. pharmacopeial monographs for amoxicillin tablets,^31^ capsules,^32^ and oral suspensions.^33^ For each sample of tablets, six units were randomly selected and dissolved in pH 5.0 acetate buffer (0.01 M) using magnetic stirring for at least 30 minutes to yield a concentration of approximately 0.8 mg/mL of anhydrous amoxicillin. Capsules (250 mg and 500 mg) were prepared by randomly selecting 10 units and weighing the contents minus the capsule shell, and a mass equivalent to approximately 200 mg anhydrous amoxicillin was initially sonicated and then magnetically stirred. Oral suspensions were reconstituted, and a 15.0-mL sample was diluted with pH 5.0 acetate buffer (0.01 M) to yield a concentration of approximately 0.8 mg/mL of anhydrous amoxicillin, which was dissolved using sonication and magnetic stirring.

Standard solutions were prepared in pH 5.0 acetate buffer (0.01 M) using amoxicillin trihydrate powder (Acros Organics) with a separate quality control standard (0.7 mg/mL amoxicillin anhydrous) prepared using certified reference material purchased from Sigma Aldrich. All sample and standard solutions were filtered with a 0.45-μm regenerated cellulose syringe filter (Cole-Parmer) and analyzed by high-performance liquid chromatography (HPLC). The chromatographic system consisted of a Shimadzu Nexera-i LC2040C (Shimadzu Corporation, Kyoto, Japan) with the autosampler set at 15°C and a column oven temperature of either 40°C for the tablet and capsule samples or 35°C for the analysis of the oral suspensions. The analytical column used was a Waters (Milford, MA) XSelect HSS T3 5 μm (4.6 × 250 mm), and an injection volume of 5 μL was used. The pre-mixed isocratic mobile phase consisted of 2.5% (volume/volume) acetonitrile (HPLC grade, Ajax Finechem, Sydney, Australia) in 0.01 M acetate buffer of pH 5.0 (sodium acetate, AR grade, Ajax Finechem; glacial acetic acid, AR grade, Lab-Scan). The flow rate and wavelength of detection were 1.0 mL/min and 271 nm, respectively. The method was validated (Supplemental Table S1) for linearity, precision, accuracy, and specificity in the presence of excipients and the forced degradation products generated by exposure of amoxicillin to acidic, basic, and oxidative stress conditions as per International Council for Harmonisation guideline Q2(R1).^34^

### Statistics.

Fisher’s exact test was performed using SPSS version 25 (IBM, Armonk, NY) with significance set at two-tailed *P* < 0.05.

## RESULTS

### Overview of collection sites and samples.

The two surveys were conducted 5 months apart, and the collection sites represented 94% (2018) and 91% (2019) of licensed medicine outlets in the NCD (Table 1). Only one site had no functioning air conditioning in 2018, and this was noted again during collection in 2019.

**Table 1:** Summary of licensed pharmaceutical outlets visited and samples collected

Survey	October 2018	March/April 2019	Total in the National Capital District
Retail pharmacies	23	22	24
Private health facilities	4	4	4
Public health facilities	5	4	5
Area medical stores	0	1	1
Total sites	32	31	34
	Number of samples		
Dosage form and strength	October 2018	March/April 2019	
Tablets (250 mg)	14	12	–
Capsules (250 mg)	21	17	–
Capsules (500 mg)	30	28	–
Suspensions (125 mg/5 mL)	21	23	–
Suspensions (250 mg/5 mL)	4	22	–
Total samples collected	90	102	–

A total of 192 samples were collected (Supplemental Table S2), and from the 135 samples whose packaging enabled identification, 22 separate manufacturers and 60 unique batch numbers were noted. The remaining samples (57) were dispensed in plastic bags or bottles and lacked information to indicate the manufacturer or batch number. An analysis of the manufacturers named on the samples revealed two trends. First, a number of the manufacturers were well represented in both surveys, with numerous batch numbers and multiple dosage forms collected. It was noted that within the retail sector, products from a selected number of manufacturers were exclusively dispensed by specific retail pharmacy chains. The second trend applied to individual dosage forms where it was observed that a large number of samples with the same batch number were collected, suggesting bulk importation of the product had occurred. In some instances, the manufacturer was found in only one of the surveys. In each of the surveys, the total number of oral dosage forms was equal, with the exception of the 250 mg/5 mL oral suspension which was underrepresented in the 2018 survey.

### Assessment of product packaging.

**Figure 1.**
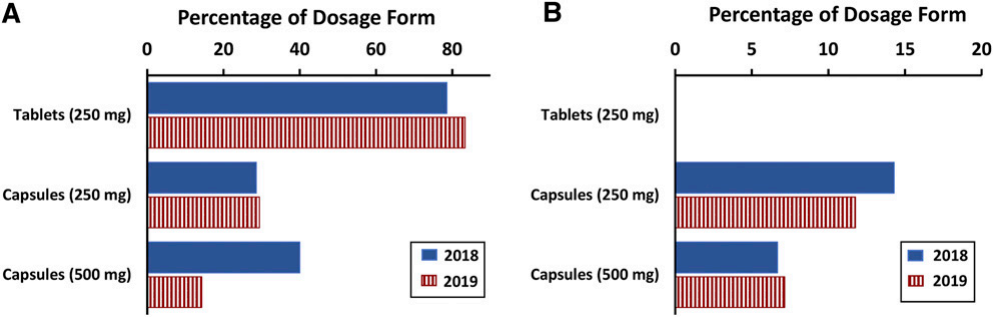
Percentage of samples dispensed as individual units in (**A**) plastic bags and (**B**) plastic bottles. This figure appears in color at www.ajtmh.org.

The amoxicillin oral dosage forms were dispensed with a variety of packing, ranging from plastic bags or bottles containing individual units (tablets/capsules) to medicines inclusive of their primary and secondary packaging. Tablets were almost exclusively dispensed as individual units in plastic bags (Figure 1A), with the exception of five samples which were obtained as blister strips in plastic bags. From the packaging, the latter were identified as dispersible tablets, and because the two identified batch numbers were collected in both 2018 and 2019, it was possible to determine the stock did not turn over during the 5 months between surveys.

The use of plastic bottles to repack was only observed for capsules (Figure 1B); however, this practice was less popular than the plastic bags (Figure 1A). The number of capsule samples (250 mg and 500 mg) dispensed without any primary packaging varied between the two surveys, with 45% in 2018 and 29% in 2019. The balance of the capsule samples were dispensed in blister strips, contained either in plastic bags or in the manufacturer’s secondary packaging. An analysis of the batch numbers from these samples identified 33 unique batches of capsules collected during the two surveys, and 24% of these were included in both surveys.

By the very nature of the product, all oral suspensions were dispensed in their primary packaging, and 80% were inclusive of the secondary packaging. In the two surveys, a total of 19 unique batch numbers were identified for the 125 mg/5 mL suspensions. Only two of these batch numbers (2/19) were collected at both times, suggesting the stock turnover of this product was the most rapid of the three dosage forms collected. In total, 12 unique batch numbers were identified from the collection of 250 mg/5 mL suspensions; however, because of the underrepresentation of this strength in the 2018 survey, the rate of stock turnover could not be assessed.

Application of the “Tool for Visual Inspection of Medicines”^30^ to identify falsified products experienced a number of limitations, as summarized in Table 2. Examination of the 192 samples collected did not identify any falsified products. All identified manufacturers had a presence on the Internet, the tamper-proof seals were intact on all suspension bottles, and where two or more identical products were collected, no discrepancies were observed in packaging appearance. Only one irregularity was noted, being a singular sample of a 125-mg/5 mL suspension collected from a health facility whose packaging stated the product was “manufactured for” a company in Asia but failed to identify the manufacturer or the country of manufacture.

**Table 2:** Limitations experienced when applying “Visual Tool for Inspection of Medicines”^30^ to examine authenticity of collected samples

Inspection criterion	Limitation
Product packaging	Medicines commonly dispensed without secondary packaging
	Tablets and capsules dispensed in plastic bags/bottles lacking information about manufacturer or batch number
	Products manufactured by same parent company but in different countries and with different packaging
	Same brand name produced by three manufacturers, packaging similar—Internet sources suggested that two manufacturers belonged to the same parent company, but this could not be confirmed
	Singular sample of product collected
Manufacturer	Australian and UK registered products do not always identify manufacturer and/or country of manufacture
Packaging unopened	Pharmacist opened product to cut blister strips and/or write instructions on inside of carton or on the bottle
Leaflet/package insert	Packaging opened by pharmacist, leaflet missing
	Australian registered products not produced with leaflet

### Content analysis and quality of reconstituted suspensions.

**Figure 2.**
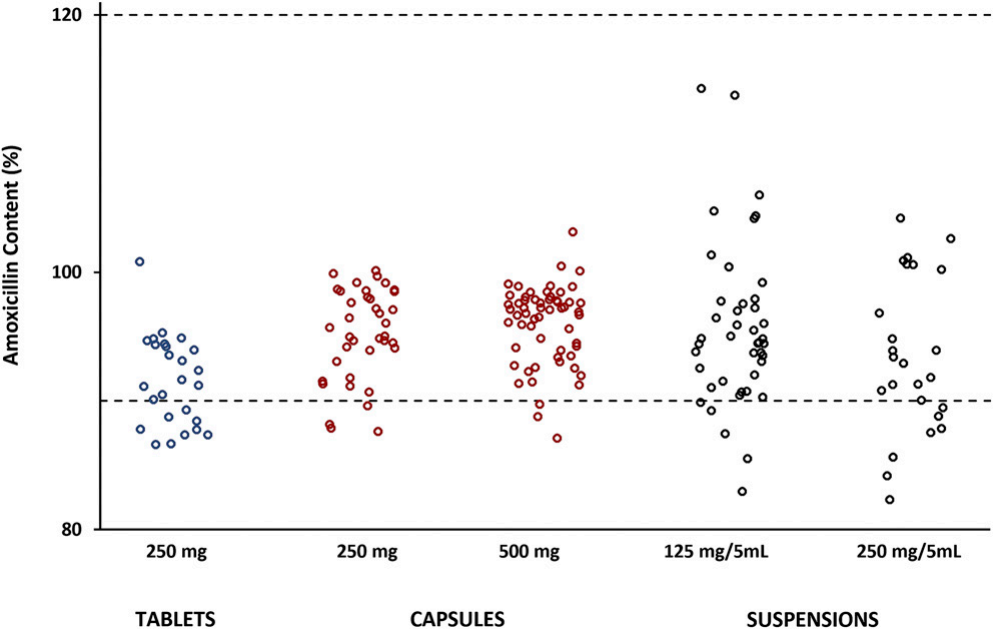
Measured amoxicillin content of oral dosage forms expressed as percentage of nominal content. Dotted lines indicate United States Pharmacopeia requirements for amoxicillin (≥ 90.0–≤ 120.0%).^31–33^ This figure appears in color at www.ajtmh.org.

All 190 samples subjected to content analysis were found to contain amoxicillin (Figure 2), with 82.3% being the lowest concentration measured and none exceeding the pharmacopeial maximum of 120.0%.^31–33^ Half (50%, 7/14) of the tablet samples collected in 2018 were found to contain substandard amounts of amoxicillin. One failed sample was obtained from a public health facility, whereas the remainder were exclusively collected from pharmacies belonging to the same retail chain. The latter had identical physical properties (color, shape, size, and coating) and were dispensed in plastic bags, suggesting they may have been imported in bulk. In the follow-up survey of 2019, only one pharmacy still dispensed this batch of tablets, indicating stock turnover may have been slower at that location. Tablets collected in 2019 showed an improvement in the number of samples (83%, 10/12) meeting pharmacopeial content requirements; however, it was discovered that 42% (5/12) were badly broken following shipment for analysis. These samples had been dispensed in plastic bags and were found to effervesce when added to an aqueous solution, suggesting they were dispersible tablets. They were dispensed exclusively by outlets belonging to a retail chain, and all shared the same physical attributes (color, shape, size, coating, and smell). Analysis of the unbroken tablets found they contained appropriate amounts of amoxicillin; however, breakage is indicative of a lack of physical stability and from a patient perspective would result in the loss of confidence in the medicine.

Capsules (250 mg and 500 mg) had the lowest incidence of substandard amoxicillin content of the dosage forms collected (Figure 2). A total of seven samples failed pharmacopeial requirements in the two surveys, with all having been dispensed in the manufacturer’s primary packaging. Five were collected in 2018 from retail pharmacies, and all were produced by the same manufacturer, but marketed under two brand names. In this group, three unique batch numbers were identified, and in 2019, one sample from a failed batch was again collected. The other failed sample from 2019 was obtained from a public health facility and was a brand not previously seen. Product packaging claimed the manufacturer was WHO Good Manufacturing Practices (GMP) certified; however, it was noted that the color of the powder varied between individual capsules (Supplemental Figure S1A), suggesting poor blending during manufacture.

Reconstitution of the oral suspensions in accordance with the manufacturers’ instructions was problematic, with many failing to produce a homogenous mixture. This was found to be directly manufacturer/batch-related, with the following problems observed: 1) the powder had poor wettability that quickly clumped on contact with water and excessive shaking could not disperse the powder evenly; 2) reconstitution appeared successful but hard, and irregularly sized gritty particles were present; and 3) when shaken, a more viscous component adhered to the bottle surface. The high failure rates of 125 mg/5 mL suspensions (48%, 11/23) and 250 mg/5 mL suspensions (59%, 13/22) in the 2019 survey (Supplemental Table S3) were directly related to the greater incidence of problematic brands and/or batch numbers.

Analysis of the reconstituted suspensions for amoxicillin content was complicated when poor homogeneity was encountered despite efforts to reduce the size of aggregated material through the use of laboratory equipment. Only four samples across both surveys were found to contain substandard amounts of amoxicillin and be homogenous, with two having the lowest measured drug content of this study (82.3% and 82.9%). The four substandard samples had different batch numbers, and three manufacturers were represented. Most suspensions failing content requirements in this study were not able to achieve homogeneity on reconstitution. Although the repeatability of sampling for homogeneous and nonhomogeneous suspensions was found to be comparable (1.6% and 1.7% relative SD, respectively, for *n* = 3 determinations), it is acknowledged that sampling may have selectively excluded aggregated material. Consequently, the analysis cannot definitively indicate if the dry powder contained substandard drug content before reconstitution; however, the values shown in Figure 2 may be indicative of the amoxicillin dose a patient might receive.

Of the 190 samples analyzed, only two (both 125 mg/5 mL suspensions) were tested after their expiry dates, having been dispensed 1 month before their expiry and analyzed 1 month after the date had elapsed. Both products passed pharmacopeial content requirements.

### Miscellaneous quality issues.

Blister strips of capsules (250 mg) collected from a private health facility and dispensed inclusive of the secondary packaging were found to have been cut lengthwise, compromising the seal on many blisters. Only capsules from undamaged blisters were selected for content analysis, which revealed the drug content met pharmacopeial requirements. However, the capsule material was not free-flowing and was expressed from the shells as a hardened powder (Supplemental Figure S1B) that required crushing to enable sampling, with the potential to affect dissolution and bioavailability for patients.

Two samples of 500 mg capsules sharing the same batch number were found to have leaked powder that adhered to the clear surface of the manufacturer’s primary packaging, and investigation revealed this was caused by damage to the capsule body (Supplemental Figure S1C) during production. These capsules were not analyzed, with the remaining capsules found to contain the requisite amount of amoxicillin.

### Risk factors associated with substandard content.

No association was found between those samples with substandard amoxicillin content and the time of year sampled (*P* = 0.54) or the practice of dispensing tablets and capsules in plastic bags versus primary packaging (*P* = 0.41). The type of dosage form (tablet, capsule, and suspension) was however found to be significant (*P* = 0.002). When the retail pharmacies were categorized by their dominance of the local market (“chain x,” “chain y,” and “other”), an association with substandard content was likewise identified (*P* = 0.002). The limited sample size obtained for public and private health facilities (Supplemental Table S4) prevented statistical analysis.

## DISCUSSION

In this study, amoxicillin oral dosage forms from licensed medicine outlets in the NCD of PNG were collected at two time periods and analyzed to determine their amoxicillin content. From the data, two risk factors were identified as contributing to the poor quality of these products in the region. No falsified medicines were identified in the 192 samples collected; however, the inspection process was compromised by the volume of products not dispensed with the manufacturer’s secondary packaging. This observation is consistent with a 2011 nationwide survey of public health facilities,^26^ where systematic visual inspection of collected products was not undertaken because medicines were repackaged. As these two studies are the most comprehensive performed in PNG and reported in the public domain, the true incidence of falsified medicines in PNG remains unknown. Thus, the practices of dispensing medicines without their secondary packaging, and repacking loose tablets and capsules in plastic bags are enablers for falsified medicines to pass into the PNG community. It should be noted that scrutiny of product packaging is one of the few tools available to the general public to determine whether their medicines are falsified.^35^

Of the 190 samples analyzed for amoxicillin content, 15% (28/190) failed pharmacopeial requirements. We identified two risk factors associated with substandard content, namely, the type of dosage form (*P* = 0.002) and the retail pharmacy chain (*P* = 0.002). The significance of the dosage form was unsurprising, given the low failure rate of capsules and the higher failure rate of tablets and suspensions (Figure 2). The second risk factor can be explained by the fact that two retail pharmacy chains dominate the market in PNG. Substandard samples collected from the retail sector were almost exclusively (86%, 19/22) obtained from one of the major chains, and 47% of those failed products (9/19) were produced by two manufacturers. The implications of this finding are significant to the Western Pacific area, as in addition to importing medicines into PNG for domestic use, a number of wholesalers subsequently export products to other pacific countries.

With a hot and humid climate, it was considered that the incidence of substandard amoxicillin products in the NCD may be linked to the date of sample collection (before and after summer), and to the practice of dispensing tablets and capsules from bulk containers into plastic bags. In both cases, no statistically significant associations were identified (*P* = 0.54 and *P* = 0.41, respectively). This result is supported by the observation that all registered medicine outlets, except one, had functioning air conditioning, and implies that the amoxicillin products had been stored appropriately during transport to local outlets. Similarly, Hetzel et al.^26^ concluded that conditions during transport and in storage facilities did not contribute to the incidence of poor-quality medicines in public health facilities across PNG. It should be noted, however, that once dispensed from medicine outlets, repackaged tablets and capsules are stored in the home environment where air conditioning is a rarity. As reported by Naidoo et al.,^21^ repackaged capsules stored under hot and humid conditions can experience high rates of amoxicillin degradation. Figure 2 demonstrates that many tablet and capsule samples contained 90–95% amoxicillin, and thus there is the potential for a medicine dispensed to a patient to become substandard (< 90%) before the course of antibiotics has been completed.

Previous studies of medicine quality in PNG have identified a high incidence of substandard/falsified products in the supply chains of registered pharmacies and health facilities. Purchased in the capital city of Port Moresby in 2009, Nair et al.^27^ reported that all (100%) samples of amoxicillin (*n* = 8) and amodiaquine (*n* = 6) failed content uniformity, and at least three (3) counterfeit (falsified) products were identified. Similarly, a 2010 report^36^ identified 100% of artemether (*n* = 27) and 50% of artesunate (*n* = 16) samples failed content analysis, with collection occurring in the NCD from pharmacies and a public hospital. The 2011 nationwide sampling program of public health facilities (60 providers, 11 medicines, and 360 samples)^26^ found 10% of samples failed to meet pharmacopeial content requirements. In recent years, a number of WHO-supported initiatives have been implemented to improve medicine quality in PNG, including strengthening of the national pharmaceutical legislation^37^ and opening of a Medicines Quality Control Laboratory (November 2017),^38^ and in August 2019, it was announced that all medicines entering the country must undergo registration via the Medicines Market Authorization System.^39^

The failure rate identified in this study (15%, 28/190) is concerning, particularly when interpreted in the context of antimicrobial resistance. Mathematical models simulating the development of antibiotic resistance predict the greatest risk occurs in the subtherapeutic window, when drug levels are too low to kill resistant strains, but sufficiently high to kill susceptible strains.^40–42^ Models have also indicated that the likelihood of a patient hosting new resistant strains is higher for an individual adhering to their treatment regimen but taking substandard antibiotics than if the individual failed to complete the course of a quality medication.^42^ For a community such as the NCD (land area 240 km^2^, estimated population at 2011 census of 364,125^43^), which is geographically isolated and not linked by road to most of the rest of the country,^22^ the implications are significant. The results of this study have shown that the practice of importing medicines in bulk, and often from the same batch, can significantly increase the incidence of substandard amoxicillin if that batch is of poor quality. Thus, at any one point in time, a high proportion of patients in the NCD could potentially be exposed to subtherapeutic levels of amoxicillin. Furthermore, should treatment be unsuccessful with a single course, it seems likely that the patient would receive a repeat course of the same drug product, possibly acquired from the same medicine outlet.

The prevalence of substandard amoxicillin dosage forms in the NCD identified by this study (15%, 28/190) indicates further improvements to medicine quality in PNG are necessary to improve patient outcomes and combat the rise of antibiotic resistance. This could be achieved through utilization of the reforms PNG has introduced in recent years. Specifically, the data indicate that the testing of bulk medicines, particularly those with the same batch number, should be prioritized by the Medicines Quality Control Laboratory. Furthermore, it is recommended that the newly introduced Medicines Marketing Authorization System take remedial action against manufacturers who repeatedly supply substandard drug products.

## LIMITATIONS OF THE STUDY

This project sought to collect oral dosage forms of amoxicillin from licensed medicine outlets and subsequently measure the drug content. Safety considerations excluded sampling from unlicensed vendors (e.g., market stalls) and consequently the reported incidences of substandard and falsified amoxicillin products for the NCD may underrepresent the true level in the community. Furthermore, the study did not assess the dissolution rate, which is an indicator of drug bioavailability and key quality criterion regulated by pharmacopeias.

## CONCLUSION

Substandard and falsified medicines are a global scourge, with low- to middle-income countries being less equipped to monitor and regulate the quality of medicines in their domestic supply chains. With growing concern about the escalating rates of antimicrobial resistance, the contribution of poor quality medicines is increasingly being examined. We have presented a unique study detailing the quality of oral dosage forms of amoxicillin in PNG at two time periods and identified risk factors, which if addressed can potentially reduce the incidence of antimicrobial resistance and improve health outcomes for people in PNG.

## Supplemental tables and figure

Supplemental materials
