# Supplementary material for: Prevalence of Substandard Amoxicillin Oral Dosage Forms in the National Capital District of Papua New Guinea
Source: Am J Trop Med Hyg. 2021 May 17;105(1):238–44. doi: 10.4269/ajtmh.20-1570 (PMC8274755; doi:10.4269/ajtmh.20-1570)
Supplement: Supplementary file 1 [file tpmd201570.SD1.pdf]

SUPPLEMENTAL MATERIAL

TABLE S1. Validation of HPLC method for the determination of amoxicillin (calibration range 0.2 to 1.2 mg/mL amoxicillin anhydrous)

**Statistic**[b]	**Concentration**[a] **(mg/mL)** 0.202	0.821	1.262
Mean measured (mg/mL)	0.198	0.821	1.251
% Relative standard deviation	0.286	0.0930	4.622 E-05
% Relative error	2.145	0.0406	0.845
Typical regression equation	Y = (825149.8)X + (1646.2)		
Linearity > 0.9999			

[a] Prepared using certified reference material (see Methods for details)

[b] (n=3) replicate determinations

TABLE S2. List of amoxicillin dosage forms examined in this study. (separate file)

TABLE S3. Percentage of suspensions that were non-homogeneous upon reconstitution.

**Strength**	**Collection Date** Oct 2018	Mar/Apr 2019
125mg/5mL	14.3%	47.8%
250mg/5mL	25.0%	59.1%
Total collection: 40.0% (28/70)		

TABLE S4 Percentage of samples failing content requirements by category of medicine outlet.

**Outlet Category**	**Number of Samples** Analyzed	Failed
Retail pharmacy	148	22 (14.9%)
Private health facility	17	2 (11.8%)
Public health facility[a]	25	4 (16.0%)

**TOTAL**	**190**	**28 (14.7%)**

[a] Includes samples collected from Area Medical Store

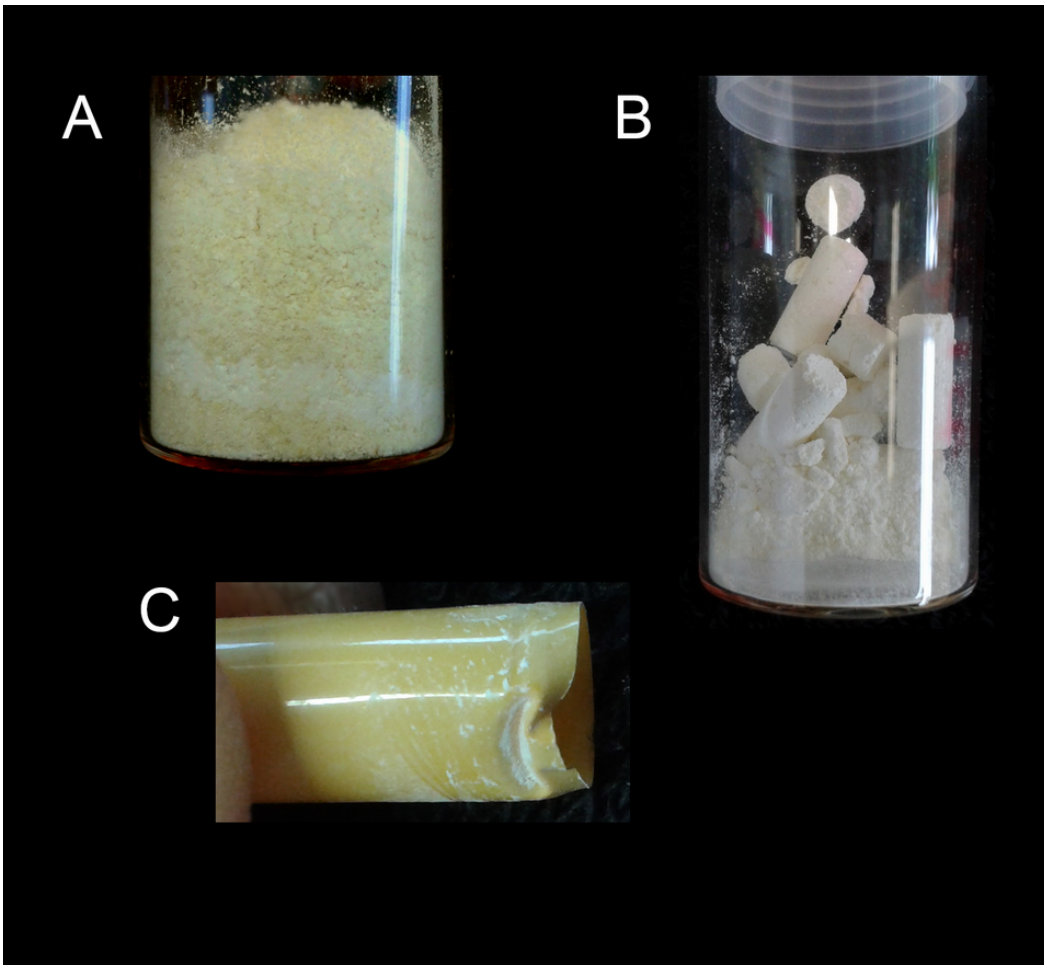

FIGURE S1. Other quality issues identified from capsule samples. A) powder collected from ten shells showing variation in color of fill material, B) material not free-flowing and expressed as a hardened slug, and C) capsule shell damaged during production causing powder leakage into blister well. Photographs not to scale.
