# Supplementary material for: Prevalence of Substandard Amoxicillin Oral Dosage Forms in the National Capital District of Papua New Guinea
Source: Am J Trop Med Hyg. 2021 May 17;105(1):238–44. doi: 10.4269/ajtmh.20-1570 (PMC8274755; doi:10.4269/ajtmh.20-1570)
Supplement: Supplementary file 2 [file tpmd201570.SD2.pdf]

TABLE S2 List of amoxicillin oral dosage forms examined in this study.

Dosage Form	Strength	Manufacturer (de-identified[a])	Brand name (de-identified[a])	Country of Manufacture	Batch Number	Packaging	Shelf life[b] remaining at collection [months]
Capsule	250 mg	M 1	B 1	India	170181	primary + secondary	3
Capsule	250 mg	M 2	B 2	not stated	2841644	primary + secondary	14
Capsule	250 mg	M 2	B 2	not stated	2841644	primary + secondary	14
Capsule	250 mg	M 2	B 2	not stated	2841644	primary + secondary	14
Capsule	250 mg	M 2	B 2	India	2841644	primary + secondary	9
Capsule	250 mg	M 2	B 2	not stated	2841644	primary + secondary	10
Capsule	250 mg	M 2	B 2	not stated	2934956	primary + secondary	26
Capsule	250 mg	M 2	B 2	not stated	2934956	primary + secondary	26
Capsule	250 mg	M 2	B 2	not stated	2934956	primary + secondary	22
Capsule	250 mg	M 2	B 2	not stated	2934956	primary + secondary	22
Capsule	250 mg	M 2	B 2	not stated	2934956	primary + secondary	21
Capsule	250 mg	M 2	B 2	not stated	2934956	primary + secondary	22
Capsule	250 mg	M 2	B 2	not stated	2934956	primary + secondary	22
Capsule	250 mg	M 3	B 3	India	CLABV0003	primary + secondary	3
Capsule	250 mg	M 3	B 3	India	CLABV0003	primary + secondary	3
Capsule	250 mg	M 4a*	B 4	not stated	HAFAU	primary + secondary	13
Capsule	250 mg	M 5a**	B 5	India	S797087	blister strip in plastic bag	20
Capsule	250 mg	M 5a**	B 5	India	S797087	blister strip in plastic bag	20
Capsule	250 mg	M 5a**	B 5	India	S797215	primary + secondary	21
Capsule	250 mg	M 5a**	B 6	India	S797215	primary + secondary	25
Capsule	250 mg	M 5a**	B 6	India	S797215	primary + secondary	25
Capsule	250 mg	M 6	B 5	India	EBC180014	primary + secondary	25
Capsule	250 mg	not documented	not documented	repacked		plastic bag	?
Capsule	250 mg	not documented	not documented	repacked		plastic bottle	?
Capsule	250 mg	not documented	not documented	repacked		plastic bottle	?
Capsule	250 mg	not documented	not documented	repacked		plastic bag	?
Capsule	250 mg	not documented	not documented	repacked		plastic bag	?
Capsule	250 mg	not documented	not documented	repacked		plastic bag	?
Capsule	250 mg	not documented	not documented	repacked		plastic bottle	?
Capsule	250 mg	not documented	not documented	repacked		plastic bag	11
Capsule	250 mg	not documented	not documented	repacked		plastic bag	9
Capsule	250 mg	not documented	not documented	repacked		plastic bottle	?
Capsule	250 mg	not documented	not documented	repacked		plastic bag	11
Capsule	250 mg	not documented	not documented	repacked		plastic bag	5
Capsule	250 mg	not documented	not documented	repacked		plastic bottle	?
Capsule	250 mg	not documented	not documented	repacked		plastic bag	?
Capsule	250 mg	not documented	not documented	repacked		plastic bag	6
Capsule	250 mg	not documented	not documented	repacked		plastic bag	?
Capsule	500 mg	M 1	B 1	India	170979	primary + secondary	9
Capsule	500 mg	M 1	B 1	India	171336	primary + secondary	12
Capsule	500 mg	M 3	B 3	not stated	CMBBV0008	primary + secondary	27
Capsule	500 mg	M 3	B 3	not stated	CMBBV0008	primary + secondary	27
Capsule	500 mg	M 3	B 3	not stated	CMBBV0009	primary + secondary	24
Capsule	500 mg	M 4b*	B 4	France	6A8B	primary + secondary	13
Capsule	500 mg	M 5a**	B 5	India	0218P002	blister strip in plastic bag	26
Capsule	500 mg	M 5a**	B 5	India	0218P002	blister strip in plastic bag	?
Capsule	500 mg	M 5a**	B 5	India	S797214	primary + secondary	25
Capsule	500 mg	M 5a**	B 5	India	S797214	primary + secondary	21
Capsule	500 mg	M 5a**	B 6	India	S797214	primary + secondary	25
Capsule	500 mg	M 5a**	B 6	India	S797214	primary + secondary	25
Capsule	500 mg	M 6	B 5	India	EBC180001	primary + secondary	26

Capsule	500 mg	M 6	B 5	India	EBC180001	primary + secondary	26
Capsule	500 mg	M 6	B 5	India	EBC180007	primary + secondary	28
Capsule	500 mg	M 6	B 5	India	EBC180007	primary + secondary	28
Capsule	500 mg	M 6	B 5	India	EBC180033	primary + secondary	29
Capsule	500 mg	M 6	B 5	India	EBC180033	primary + secondary	29
Capsule	500 mg	M 6	B 5	India	EBC180033	primary + secondary	28
Capsule	500 mg	M 6	B 5	India	EBC180033	primary + secondary	28
Capsule	500 mg	M 6	B 5	India	EBC180055	primary + secondary	32
Capsule	500 mg	M 6	B 5	India	EBC180055	primary + secondary	33
Capsule	500 mg	M 6	B 5	India	EBC180055	primary + secondary	33
Capsule	500 mg	M 6	B 5	India	EBC180055	primary + secondary	33
Capsule	500 mg	M 7	Amoxicillin capsules	not stated	T7027010	primary + secondary	27
Capsule	500 mg	M 7	Amoxicillin capsules	not stated	T7027010	primary + secondary	23
Capsule	500 mg	M 7	Amoxicillin capsules	repacked	T7027010	blister strip in plastic bag	22
Capsule	500 mg	M 8	B 7	Philippines	7D095A	primary + secondary	7
Capsule	500 mg	M 9	Amoxicillin capsules	China	1812149	blister strip in plastic bag	34
Capsule	500 mg	M 10	Amoxicillin capsules	China	1806515	primary + secondary	32
Capsule	500 mg	M 10	Amoxicillin capsules	China	1806515	primary + secondary	28
Capsule	500 mg	M 11	Amoxicillin	repacked	217014	blister strip in plastic bag	18
Capsule	500 mg	M 11	Amoxicillin	repacked	217014	blister strip in plastic bag	13
Capsule	500 mg	M 12	B 9	Austria	HS8599	primary + secondary	9
Capsule	500 mg	M 12	B 9	Austria	HZ1977	primary + secondary	15
Capsule	500 mg	M 13	Amoxicillin capsules	India	SB016K02	blister strip in plastic bag	18
Capsule	500 mg	not documented	B 7	repacked		plastic bag	?
Capsule	500 mg	not documented	B 8	repacked	36685	plastic bottle	?
Capsule	500 mg	not documented	not documented	repacked	8D105A	blister strip in plastic bag	38
Capsule	500 mg	not documented	not documented	repacked		blister strip in plastic bag	4
Capsule	500 mg	not documented	not documented	repacked		plastic bag	?
Capsule	500 mg	not documented	not documented	repacked		plastic bag	?
Capsule	500 mg	not documented	not documented	repacked		plastic bag	16
Capsule	500 mg	not documented	not documented	repacked		plastic bag	?
Capsule	500 mg	not documented	not documented	repacked		plastic bag	12
Capsule	500 mg	not documented	not documented	repacked		plastic bag	?
Capsule	500 mg	not documented	not documented	repacked		plastic bag	26
Capsule	500 mg	not documented	not documented	repacked		plastic bag	26
Capsule	500 mg	not documented	not documented	repacked		plastic bag	?
Capsule	500 mg	not documented	not documented	repacked		plastic bag	?
Capsule	500 mg	not documented	not documented	repacked		plastic bag	29
Capsule	500 mg	not documented	not documented	repacked		plastic bottle	?
Capsule	500 mg	not documented	not documented	repacked		plastic bag	6
Capsule	500 mg	not documented	not documented	repacked		plastic bottle	?
Capsule	500 mg	not documented	not documented	repacked		plastic bag	?
Capsule	500 mg	not documented	not documented	repacked		plastic bottle	?
Capsule	500 mg	not documented	not documented	repacked		plastic bag	?
Capsule	500 mg	not documented	not documented	repacked		plastic bag	22
Suspension	125mg/5mL	M 1	B 1	India	170661	primary	7
Suspension	125mg/5mL	M 1	B 1	India	170663	primary	7
Suspension	125mg/5mL	M 1	B 1	India	810259	primary	11
Suspension	125mg/5mL	M 1	B 1	India	810426	primary	13
Suspension	125mg/5mL	M 1	B 1	India	810426	primary	12
Suspension	125mg/5mL	M 1	B 1	India	810427	primary	12
Suspension	125mg/5mL	M 1	B 1	India	810427	primary	13
Suspension	125mg/5mL	M 2	B 2	not stated	2922388	primary + secondary	1
Suspension	125mg/5mL	M 2	B 2	repacked	2923850	primary	1
Suspension	125mg/5mL	M 4a*	B 4	not stated	HAIJZ	primary + secondary	26
Suspension	125mg/5mL	M 4b*	B 4	France	3Y5E	primary + secondary	20

Suspension	125mg/5mL	M 4b*	B 4	France	3Y5E	primary + secondary	19
Suspension	125mg/5mL	M 4b*	B 4	France	3Y5E	primary + secondary	20
Suspension	125mg/5mL	M 4b*	B 4	France	3Y5E	primary + secondary	20
Suspension	125mg/5mL	M 4b*	B 4	France	3Y5E	primary + secondary	20
Suspension	125mg/5mL	M 4b*	B 4	France	3Y5E	primary + secondary	20
Suspension	125mg/5mL	M 4b*	B 4	France	3Y5E	primary + secondary	20
Suspension	125mg/5mL	M 5b**	B 5	India	0329P001	primary + secondary	37
Suspension	125mg/5mL	M 6	B 5	India	EBD180009	primary + secondary	28
Suspension	125mg/5mL	M 6	B 5	India	EBD180009	primary + secondary	28
Suspension	125mg/5mL	M 6	B 5	India	EBD180009	primary + secondary	28
Suspension	125mg/5mL	M 6	B 5	India	EBD180009	primary + secondary	28
Suspension	125mg/5mL	M 6	B 5	India	EBD180009	primary + secondary	28
Suspension	125mg/5mL	M 6	B 5	India	EBD180009	primary + secondary	23
Suspension	125mg/5mL	M 6	B 5	India	EBD180009	primary + secondary	24
Suspension	125mg/5mL	M 6	B 5	India	EBD180010	primary + secondary	29
Suspension	125mg/5mL	M 6	B 5	India	EBD180010	primary + secondary	29
Suspension	125mg/5mL	M 6	B 5	India	EBD180010	primary + secondary	29
Suspension	125mg/5mL	M 6	B 5	India	EBD180010	primary + secondary	29
Suspension	125mg/5mL	M 6	B 5	India	EBD180010	primary + secondary	29
Suspension	125mg/5mL	M 6	B 5	India	EBD180010	primary + secondary	29
Suspension	125mg/5mL	M 8	B 7	Philippines	7L014A	primary + secondary	14
Suspension	125mg/5mL	M 8	B 7	Philippines	7L014A	primary + secondary	10
Suspension	125mg/5mL	M 8	B 7	Philippines	7L014A	primary + secondary	10
Suspension	125mg/5mL	M 8	B 7	Philippines	7L015A	primary + secondary	14
Suspension	125mg/5mL	M 8	B 7	Philippines	7L015A	primary + secondary	14
Suspension	125mg/5mL	M 14	B 10	India	8402002	primary	8
Suspension	125mg/5mL	M 15	B 11	India	PD8055	primary + secondary	16
Suspension	125mg/5mL	M 15	B 11	India	PD8055	primary + secondary	16
Suspension	125mg/5mL	M 15	B 11	India	PD8088	primary + secondary	11
Suspension	125mg/5mL	M 15	B 11	India	PD8088	primary + secondary	11
Suspension	125mg/5mL	M 15	B 11	India	PD8088	primary + secondary	13
Suspension	125mg/5mL	M 16	Amoxycillin powder	China	170921	primary	23
Suspension	125mg/5mL	unknown ("for company --")	B 12	not stated	DBP008	primary + secondary	17
Suspension	250mg/5mL	M 1	B 1	India	170999	primary	5
Suspension	250mg/5mL	M 1	B 1	India	170999	primary	5
Suspension	250mg/5mL	M 1	B 1	India	810013	primary	10
Suspension	250mg/5mL	M 4a*	B 14	not stated	HAJCO	primary + secondary	24
Suspension	250mg/5mL	M 5b**	B 5	India	0228P001	primary + secondary	30
Suspension	250mg/5mL	M 5b**	B 5	India	0228P001	primary + secondary	25
Suspension	250mg/5mL	M 5b**	B 5	India	0228P001	primary + secondary	26
Suspension	250mg/5mL	M 5b**	B 5	India	0228P001	primary + secondary	26
Suspension	250mg/5mL	M 5b**	B 5	India	0228P001	primary + secondary	26
Suspension	250mg/5mL	M 5b**	B 5	India	0228P001	primary + secondary	26
Suspension	250mg/5mL	M 5b**	B 5	India	0228P001	primary + secondary	25
Suspension	250mg/5mL	M 5b**	B 5	India	0228P001	primary + secondary	26
Suspension	250mg/5mL	M 5b**	B 5	India	0228P001	primary + secondary	26
Suspension	250mg/5mL	M 5b**	B 5	India	0228P001	primary + secondary	26
Suspension	250mg/5mL	M 5b**	B 5	India	0228P001	primary + secondary	26
Suspension	250mg/5mL	M 5b**	B 5	India	0228P001	primary + secondary	26
Suspension	250mg/5mL	M 8	B 7	Philippines	7E058A	primary + secondary	2
Suspension	250mg/5mL	M 8	B 7	Philippines	7F225A	primary + secondary	5
Suspension	250mg/5mL	M 8	B 7	Philippines	7F225A	primary + secondary	5
Suspension	250mg/5mL	M 12	B 9	Austria	GU9901	primary + secondary	14
Suspension	250mg/5mL	M 12	B 9	Austria	HF7736	primary + secondary	21
Suspension	250mg/5mL	M 12	B 9	Austria	HL0634	primary + secondary	20
Suspension	250mg/5mL	M 17	B 4	not stated	AHBHV0005	primary + secondary	22

Suspension	250mg/5mL	M 17	B 4	not stated	AHBHV0005	primary + secondary	23
Suspension	250mg/5mL	M 18	B 13	Malaysia	1850262	primary	28
Suspension	250mg/5mL	M 19	Amoxicillin Sugar Free	not stated	161530	primary + secondary	7
Tablet	250 mg	not documented	Amoxicillin Dispersible	repacked	171495	blister strip in plastic bag	25
Tablet	250 mg	not documented	Amoxicillin Dispersible	repacked	171495	blister strip in plastic bag	25
Tablet	250 mg	not documented	Amoxicillin Dispersible	repacked	171495	blister strip in plastic bag	20
Tablet	250 mg	not documented	Amoxicillin Tablets for oral suspension	repacked	PT8023	blister strip in plastic bag	14
Tablet	250 mg	not documented	Amoxicillin Tablets for oral suspension	repacked	PT8023	blister strip in plastic bag	9
Tablet	250 mg	not documented	Amoxicllin Dispersibles[c]	repacked		plastic bag	?
Tablet	250 mg	not documented	not documented	repacked		plastic bag	?
Tablet	250 mg	not documented	not documented	repacked		plastic bag	51
Tablet	250 mg	not documented	not documented	repacked		plastic bag	?
Tablet	250 mg	not documented	not documented	repacked		plastic bag	?
Tablet	250 mg	not documented	not documented	repacked		plastic bag	27
Tablet	250 mg	not documented	not documented	repacked		plastic bag	25
Tablet	250 mg	not documented	not documented	repacked		plastic bag	43
Tablet	250 mg	not documented	not documented	repacked		plastic bag	?
Tablet	250 mg	not documented	not documented	repacked		plastic bag	25
Tablet	250 mg	not documented	not documented	repacked		plastic bag[d]	?
Tablet	250 mg	not documented	not documented	repacked		plastic bag	34
Tablet	250 mg	not documented	not documented	repacked		plastic bag	?
Tablet	250 mg	not documented	not documented	repacked		plastic bag	?
Tablet	250 mg	not documented	not documented	repacked		plastic bag	?
Tablet	250 mg	not documented	not documented	repacked		plastic bag	4
Tablet	250 mg	not documented	not documented	repacked		plastic bag	24
Tablet	250 mg	not documented	not documented	repacked		plastic bag	?
Tablet	250 mg	not documented	not documented	repacked		plastic bag	22
Tablet	250 mg	not documented	not documented	repacked		plastic bag	39
Tablet	250 mg	not documented	not documented	repacked		plastic bag	21

[a] Requirement of ethics committee

[b] Calculated difference between collection date and manufacturer's expiry date. For tablets and capsules repacked into plastic bags/bottles, expiry date was taken from Pharmacist's label (when documented)

[c] As documented on pharmacist's label

[d] Silica gel satchel included in plastic bag

*** Manufacturers coded 4a and 4b are subsidiaries of same parent company**

**** Manuacturers coded 5a and 5b treated as two separate manufacturers. Names were variations of the (apparent) parent company's and contact addresses on packaging were different. Attempts to communicate with the parent company were unsuccessful.**

	**KEY**
**?**	could not calculate (expiry not on pharmacist's label)
**plastic bag**	individual tablets/capsules repacked into plastic bag
**plastic bottle**	capsules repacked into plastic bottle
**blister strip in plastic bag**	entire or cut blister strip repacked into plastic bag
**primary**	suspension supplied in bottle only
**primary + secondary**	medicine supplied with primary and secondary packaging
**not documented**	information not included on pharmacist's label
**not stated**	information not printed on secondary packaging
**Batch number intentionally left blank when it could not be identified**	
